# Failure of ocular photodynamic therapy for secondary choroidal metastasis: a case report and literature review

**DOI:** 10.18632/oncotarget.21847

**Published:** 2017-10-16

**Authors:** Rui Hua, Wenya Li, Weiwei Wu, Jun Tao, Qing Peng

**Affiliations:** ^1^ Departments of Ophthalmology, The First Affiliated Hospital of China Medical University, Shenyang, Liaoning, P.R. China; ^2^ Departments of Thoracic Surgery, The First Affiliated Hospital of China Medical University, Shenyang, Liaoning, P.R. China; ^3^ Cancer Genetics and Comparative Genomics Branch, National Human Genome Research Institute, NIH, Bethesda, Maryland, USA; ^4^ Department of Ophthalmology, The 4th People’s Hospital of Shenyang, Shenyang, Liaoning, P.R. China; ^5^ Department of Ophthalmology, Minhang Hospital, Fudan University, Shanghai, P.R. China

**Keywords:** choroidal metastasis, photodynamic therapy, deterioration, lung cancer, angiography

## Abstract

The choroid is the most common site for intraocular metastatic disease. Photodynamic therapy (PDT) can effectively destroy malignant tissue and induce anti-tumor activity. Recent publications support its use as an effective therapy for the treatment of choroidal metastases, especially in the subfoveal region, resulting in subsequent vision preservation or improvement. Here, we introduce a case of choroidal metastasis, secondary to primary lung cancer. The progression of choroidal metastasis after PDT was followed up using spectral domain optical coherence tomography with point-to-point follow-up. Unfortunately, both the choroidal metastasis and serous retinal detachment increased after PDT. Since the mechanism underlying the therapeutic effect of PDT on choroidal metastasis is still not fully understood, deeper investigations into its safety, underlying molecular mechanisms, and treatment effects are critical for further PDT clinical usage in intraocular choroidal metastases.

## INTRODUCTION

The choroid is the most common site for intraocular metastatic disease. The hematogenous dissemination of metastases from remote sites often leads to the choroidal vasculature, which, due to its high blood flow, provides a vascular avenue for tumor emboli to settle and thrive [1]. The majority of choroidal metastases originate from breast cancer in women and lung cancer in men. Unilateral, unifocal metastases are more common in lung cancer cases [1] and the median survival time for patients with choroidal metastases originating from primary lung cancer is less than 8 months [2] Traditionally, choroidal metastases are treated with local therapy, including external beam radiotherapy, brachytherapy, transpupillary thermotherapy [3], or more recently, photodynamic therapy (PDT) [4] and anti-vascular endothelial growth factor therapy (anti-VEGF) [5, 6]. Chemotherapy and hormonal therapy are other modalities, but are usually associated with a variety of systemic side effects [2]. This is not always the traditional approach, however, if systemic chemotherapy is used and if choroidal lesions grow or arise during therapy, then local modalities are also applied. Nevertheless, Maudgil, *et al.*, reported the failure of intravitreal bevacizumab in the treatment of choroidal metastasis, attributing the failure to significant exudation and the intact choriocapillaris and Bruch’s membrane [7].

PDT uses a photosensitizer, tissue oxygen, and a particular light source to trigger the photochemical chain reaction and generate reactive oxygen species, which can then interact with amino acid residues, unsaturated lipids, or nucleic acids. In this way, PDT kills fast-growing cells by the absorption and accumulation of cytotoxic reactive oxygen species in these cells. On the other hand, in cells with a normal metabolism, PDT-induced reactive oxygen species can be cleared and cause no harm. Therefore, PDT is a safe, noninvasive, outpatient procedure with the ability to precisely target choroidal lesions; it has been approved by the Food and Drug Administration for the treatment of malignancies such as esophageal, non–small cell lung, and skin cancers [2]. PDT has been introduced as a treatment for intraocular cancer due to its minimal invasiveness and low toxicity. It usually acts as an adjunct to chemotherapy and might be most appropriate for patients who require only ocular treatment [8]. Recent publications have proven that PDT is effective for choroidal metastasis, especially subfoveal choroidal metastasis. The results of these studies were vision preservation or even improvement, without any severe PDT related complications [2]. For example, Cerman, *et al.*, collected reports of 14 choroidal metastases treated with verteporfin, and all but two cases responded with decreases in tumor size [9]. In addition, Kaliki found that one patient experienced intravitreal hemorrhage [10].

Here, we present the case of a lung cancer patient with choroidal metastasis that continued to worsen after PDT. This was demonstrated using Heidelberg spectral domain optical coherence tomography (SD-OCT) with point-to-point follow up. We also attempted to investigate the underlying mechanisms. The SD-OCT data is included in this study as a possible objective measure, since ultrasound results cannot precisely measure the thickness of tiny tumors.

## CASE REPORT

A 33-year-old Asian man was referred to us for the evaluation of a mass in his right eye, which had caused a sudden blurring of vision. He had been diagnosed with lung cancer (adenocarcinoma) a week before the appointment and had not yet received any surgery or other treatment. The patient’s best-corrected visual acuity was 20/40 in the right eye and 20/20 in the left. The intraocular pressure was normal in both eyes (19 mmHg in the right and 18 mmHg in the left).

**Figure 1:**
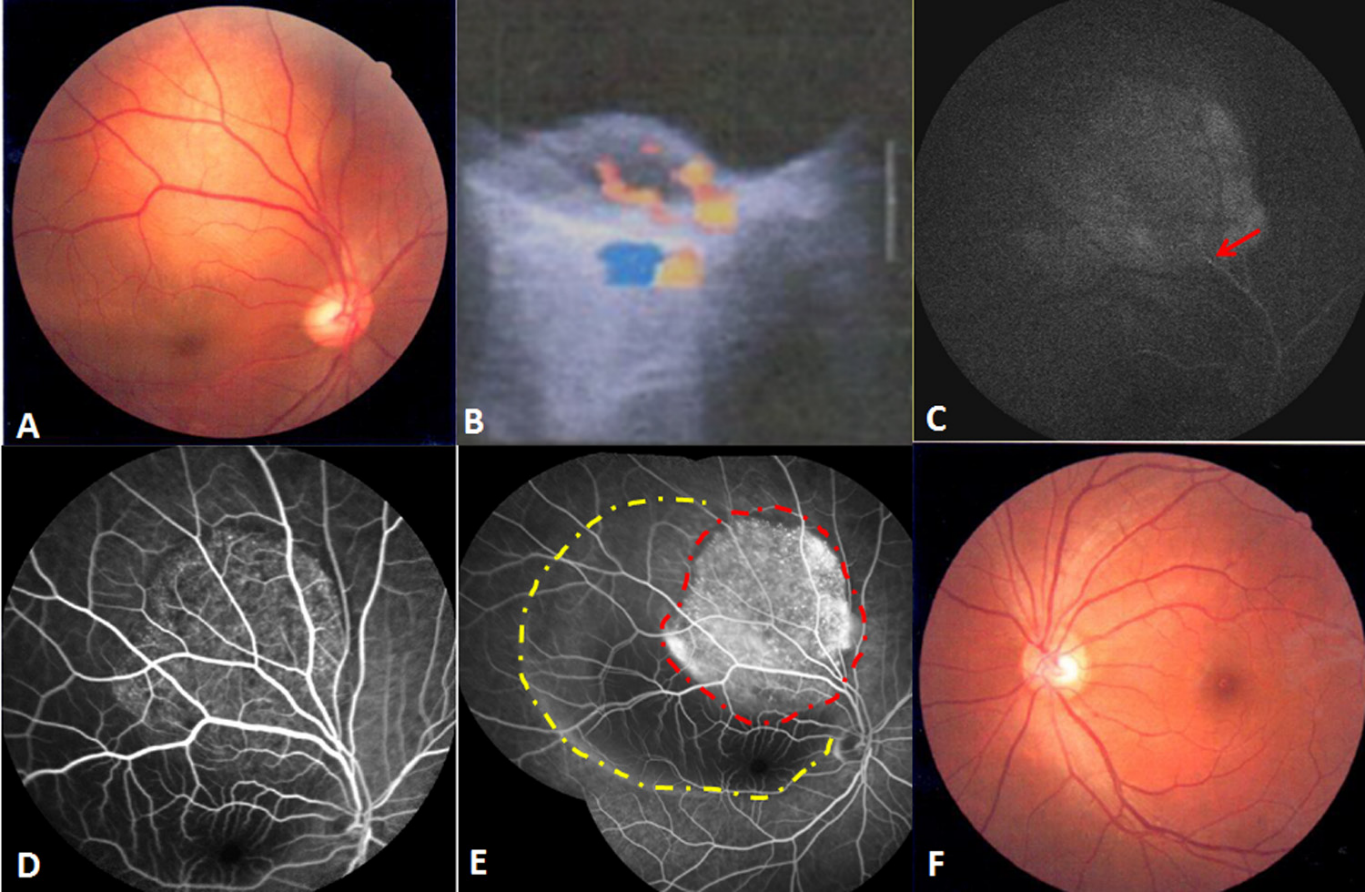
Findings at initial presentation **A**. The fundus examination of the right eye revealed a solid elevated amelanotic choroidal mass with secondary creamy yellow detachment of the retina. **B**. Ocular ultrasonic Doppler examination indicated a solid lesion occupying the choroidal space, half of which had blood pool-like blood flow, but without a classic choroidal defect. **C**. In the early FFA frame, filling of the retinal artery was observed (red arrow). **D**. In the middle FFA frame, multiple pinpoint hyperfluorescence and leakage appeared over the mass. **E**. The late FFA frame demonstrated a well-circumscribed hyperfluorescent area (red dots region) in the contour of serous retinal detachment (yellow dots region). **F**.The fundus examination of the left eye was normal.

A fundus examination of the right eye revealed a round, elevated amelanotic choroidal mass of about 5 disc diameters in size with secondary creamy yellow detachment of the retina, located just superior to the fovea. An ocular ultrasonic Doppler examination revealed a solid lesion arising from the choroid and half of the lesion had blood pool like blood flow, but without a corresponding classic choroidal defect. The tumor height on ultrasound was 4.2mm. Early fundus fluorescein angiography (FFA) frames showed multiple pinpoint hyper-fluorescence over the mass, with leakage in the later frames. The late FFA frames displayed a well-circumscribed hyperfluorescent area that filled the serous retinal detachment contour (Figure 1). Enhanced magnetic resonance imaging (MRI) scans of the affected eye showed a 0.81 x 0.38mm iso-intense nodule on both the T1 and T2 weighted images. The mass was slightly enhanced, as was the surrounding retina, after enhanced scanning. The left eye was normal.

The right eye was diagnosed with choroidal metastasis originating from lung cancer. We hesitated to use external-beam radiotherapy because of the accompanying risk of radiation retinopathy. Therefore, standard PDT with multiple overlapping spots was carried out as previously described [10]. A diode laser at 689 nm was applied 15 minutes after an intravenous injection of verteporfin (6 mg/m^2^), at an intensity of 600 mW/cm(2) for 83 seconds (50 J/cm(2)). Twelve overlapping spots (diameter: 4.3mm) were used to cover the largest tumor diameter with a 1 mm free margin, avoiding the optic disc. The need for additional PDT was determined on the basis of tumor appearance changes, increased tumor size or thickness, or recurrence of subretinal fluid [2].

**Figure 2:**
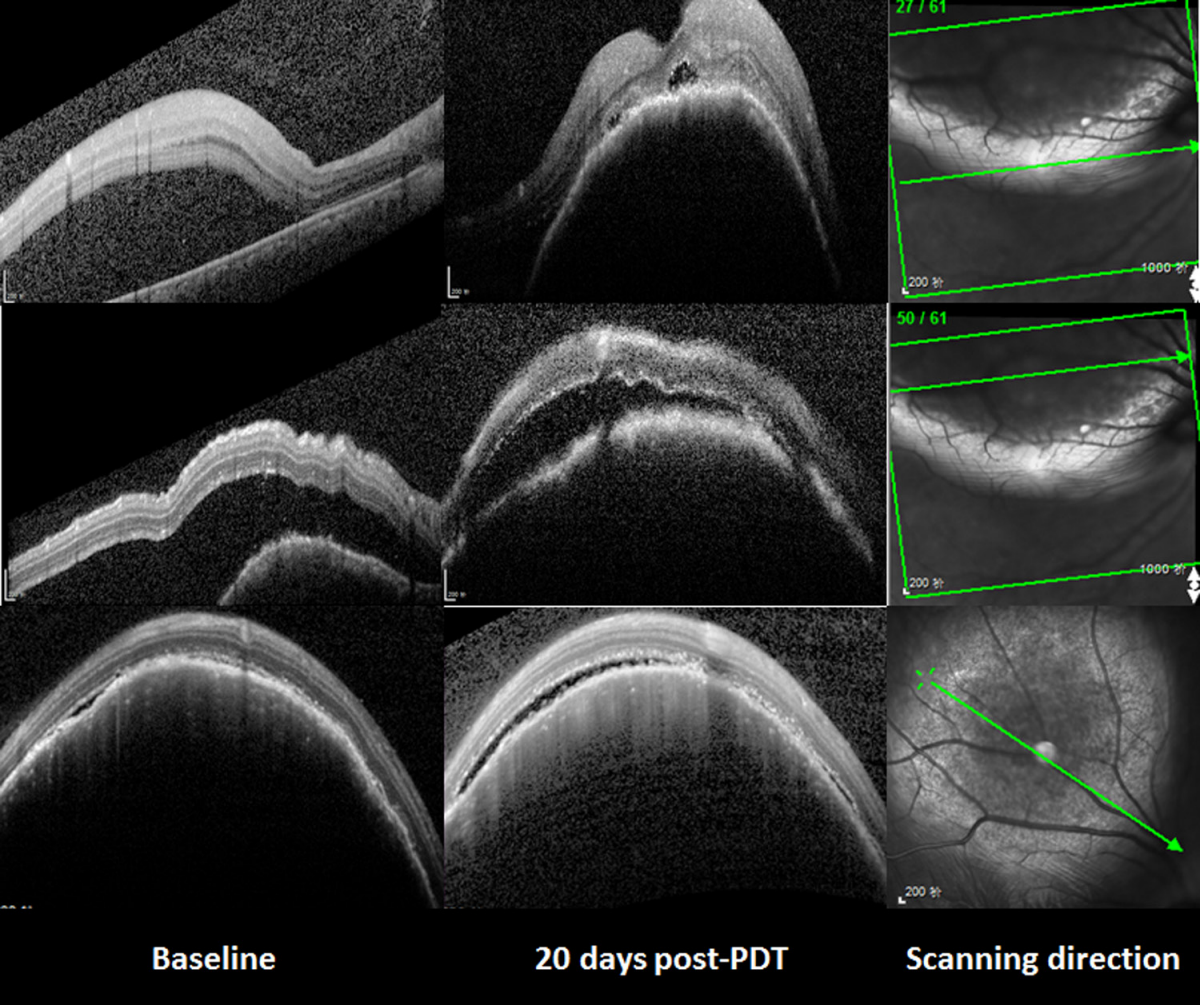
The changes of the choroidal metastasis seen with SD-OCT Compared with the baseline, both the choroidal metastasis and serous retinal detachment had increased 20 days after PDT. The area of this choroidal metastasis in the third line enlarged from 26383 (baseline) to 26851 pixels.

**Figure 3:**
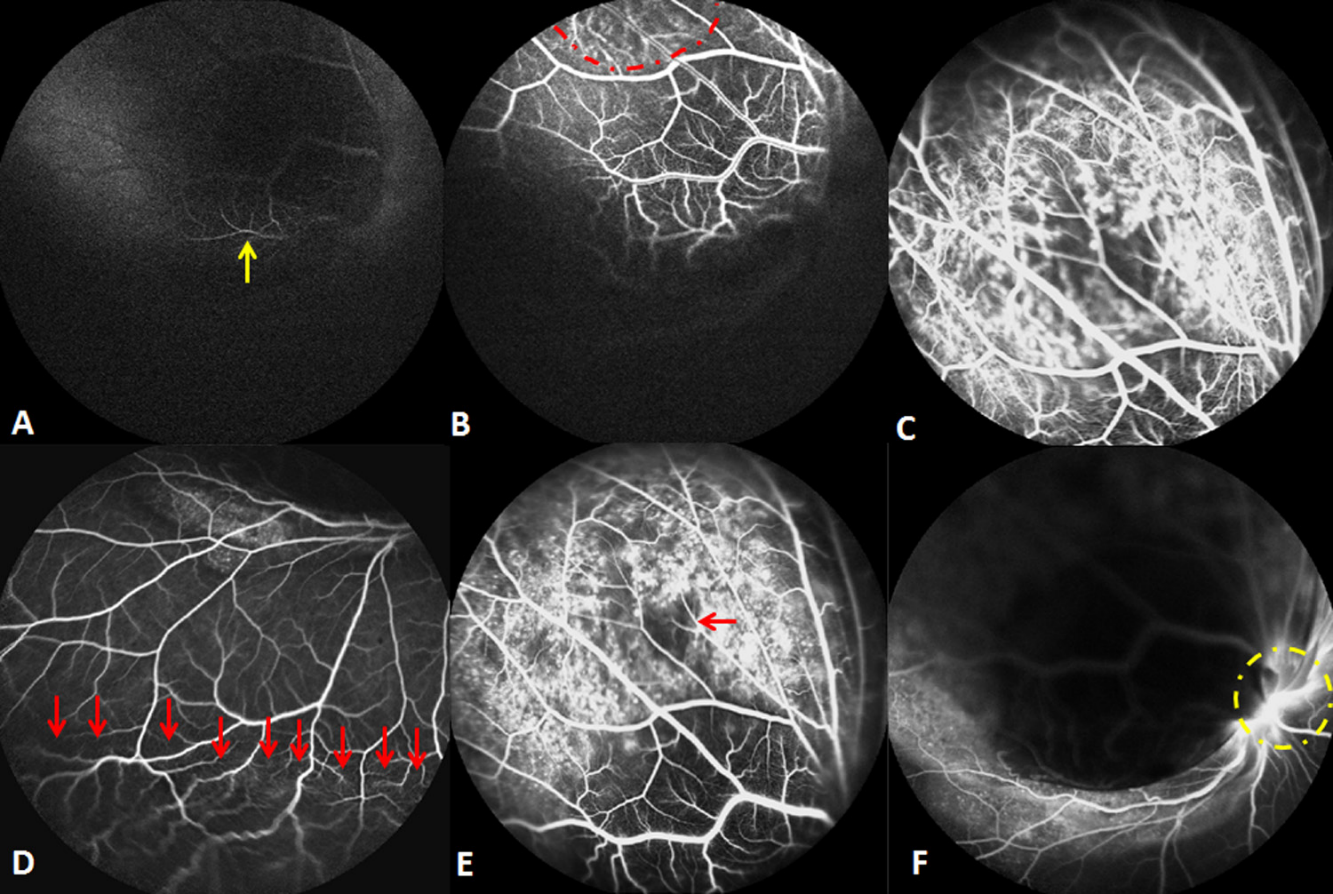
Findings six weeks after the initial PDT A. The choroidal metastasis lesion appeared to be relatively hypofluorescent in the early FFA frame, with retinal artery filling (yellow arrow). **B**. Double circulation was observed during the arterial to venous phase FFA frames (red dots region). C & D. The middle FFA frame showed the superficial dilated retinal capillaries over the mass (C) and peripheral retinal serous detachment (D. red arrows). **E**. The patched hypofluorescent necrosis region is demonstrated in the late FFA frame (red arrow). **F**. The late FFA frame shows that the mass had already involved the optic disc (yellow dots circle).

Unfortunately, both the choroidal metastasis and serous retinal detachment increased, accompanied by a further decrease of visual acuity to 2/20, all of which were assessed 20 days after PDT. SD-OCT (Spectralis HRA + OCT; Heidelberg Engineering, Heidelberg, Germany) with automatic real-time (ART) tracking was used to collect data at an acquisition rate of up to 40,000 axial scans/second. The mean number of ART Module frames for SD-OCT was 25 (range: 19-47). SD-OCT, which had previously failed to detect the choroidal metastasis in the macula, now showed a part of the choroidal metastasis at the corresponding location. In particular, the area of this choroidal metastasis in one section of the SD-OCT scanning profile was enlarged from 26383 (baseline) to 26851 pixels (as measured by Image J software, NIH, Bethesda, MD, United States) (Figure 2). In addition, six weeks after the initial PDT, FFA was repeated, in order to assess the lesion. The choroidal metastatic lesion appeared relatively hypofluorescent in the early frame, and several intralesional vessels were observed during the arterial to venous phase frames (double circulation). The superficial dilated retinal capillaries were also obvious over the mass and peripheral retinal serous detachment. Moreover, the patched hypofluorescent region of necrosis in PDT-induced choroidal metastasis was also observed in the late FFA frame. The mass involved the optic disc, as well, (Figure 3) and, when measured with FFA (Heidelberg Eye Explorer (HEYEX), version 5.3.2; Heidelberg Engineering, Germany), was shown to have increased from the initial 8.66 mm to over 10.73 mm. Unfortunately, we were not able to perform the OCT examination through the tumor site, because the choroidal metastasis had increased too heavily. During this time period, the patient refused systematic chemotherapy. He subsequently refused further treatment and defaulted further follow-up. This study adhered to the tenets of the Declaration of Helsinki, and was approved by the Medical Research Ethics Committee of The Shenyang Fourth Hospital of the People. Informed consent was obtained from the patient.

## DISCUSSION

PDT using verteporfin is believed to be effective in the management of choroidal metastasis by means of two mechanisms. Firstly, it leads directly to tumor destruction by selective cytotoxic activity towards malignant cells. Secondly, it induces intraluminal photo thrombosis in the vascular endothelial cells supplying the tumor [2]. Nevertheless, in our patient, the choroidal metastasis was aggravated by PDT and continued to grow after treatment. Similarly, two of eight tumors failed to respond to PDT, both requiring plaque radiotherapy, in a study by Kaliki [10].

According to our early observation prior to PDT, our patient’s choroidal metastasis appeared to be milder with limited activity. However, the choroidal metastasis developed rapidly with heavy leakage and its progress after PDT was different from the traditional progress of this disease.

Given the short timeline between PDT application and the subsequent enlargement of the metastatic lesion, we postulate that PDT may have worsened the metastasis through the following mechanisms. Firstly, while PDT may typically induce oxygen radicals to directly kill tumor cells through the induction of necrosis or apoptosis, thereby triggering the destruction of the tumor vasculature, it simultaneously causes DNA double-strand breaks and base mutations, potentially leading to the accumulation of gene instability [11]. This damage occurs near the photosensitizer activator, and may cause intravitreal or intraretinal hemorrhage [10]. Damage to the tumor vasculature causes hypoxia, which triggers notch singling, decreases E-cadherin expression, and increases cell migration and invasion [12]. Furthermore, hypoxia induces factor-1α to upgrade the expression of pro-inflammatory genes, therefore resulting in tumor proliferation [13]. Choroidal metastasis could also be exacerbated by laser-induced necrosis after PDT and the stimulation of VEGF expression [14].

Secondly, PDT damage to tumor cells leads to the release of molecules associated with cell death and damage, which in turn attracts leukocytes like dendritic cells and neutrophils, thereby producing an acute inflammatory response [9]. Tumor based neutrophils, however, secrete the immunoreactive neutrophil elastase, which is an independent prognostic indicator of lung cancer. Furthermore, its inhibition may be a promising preventative treatment to avoid cancer invasion and metastasis [15].

Thirdly, PDT also increases vascular permeability, causing the release of vasoactive and pro-inflammatory mediators and activating the alternative complement pathway, ultimately leading to the production of chemotactic factors and the induction of signaling cascades, which subsequently trigger cytokine secretion [9]. The vascular hyperpermeability induced by PDT facilitates focal infiltration of inflammatory mediators, as well as vascular invasion and tumor proliferation. Increasing the number of DNA double-strand breaks can promote inflammation, which induces malignant transformation and jeopardizes DNA repair, potentially resulting in increased gene instability [16].

Finally, PDT causes a local inflammatory response that includes the secretion of IL-1, TNF- alpha, and IL-6, but also causes systemic neutrophilia, increases the numbers of complement reactants, and causes a systemic release of cytokines, all of which indicate a systemic inflammatory response [9]. In our patient, the increased serious retinal detachment after PDT resulted from an increase in exudates, indicating the action of PDT on choroidal metastasis and producing a large amount of focal inflammatory substances. TNF- alpha is an important inflammatory factor that acts as a master switch in establishing an intricate link between inflammation and cancer. A wide variety of evidence has pointed to its critical role in tumor proliferation, migration, invasion, and angiogenesis [17]. In addition, IL-6 promotes tumor proliferation through the activation of the Ras/Raf/MEK pathway. Cytokines, like TNF, IL-1β, IL-4, IL-13, and TGF-β, secreted by medullary cells, can also lead to the mutation of cancer related genes, and thus, the promotion of tumor formation [18].

PDT could have facilitated the cancer cells to progress to a more malignant stage and provoked and deteriorated internal tumor reactions, further promoting the development of the choroidal metastasis. This may have occurred regardless of local therapy.

There are additional limitations of PDT for the treatment of choroidal metastasis. Lesions that are too thick might not be eligible for PDT, since the 689-nm wavelength laser may not penetrate the entire tumor. Second, additional sessions are offered in the event of subretinal fluid persistence, recurring subretinal fluid, change in tumor appearance, or increase in tumor thickness, but are at the discretion of the clinician. Third, PDT was partially effective in our patient, probably through damage of the new vessels within the choroidal metastasis. Nevertheless, since PDT does not target tumor cells, it is possible that some of these cells may survive and later cause local recurrence. Finally, this was only a case report with limited clinical experience. PDT may not be an ideal therapy for choroidal metastasis originating from early-stage lung cancer.

Theoretically, PDT can be used concomitantly with systemic chemotherapy, which, along with immunotherapy or hormone therapy, is the preferred treatment strategy in patients with bilateral, multifocal choroidal metastases. The choice of drug depends on the type of primary cancer. Fenestrated endothelium in the choriocapillaris allows drug entry into the choroidal tumors to provide effective tumor control [1]. However, no similar case has been reported in either the Science or MEDLINE databases. Alzouebi, *et al.*, reported the case of choroidal metastases secondary to breast cancer successfully regressing after the use of palliative paclitaxel and trastuzumab chemotherapy [4]. This combined therapy needs further investigation.

In the present study, we used Heidelberg SD-OCT with point-to-point follow up, in order to observe the progression of the lesion after PDT. The patient did not receive any systemic chemotherapy before or after PDT, which ensured the objective evaluation of PDT alone on choroidal metastasis. Since the mechanism underlying the therapeutic effects of PDT on choroidal metastasis is still not fully understood, deeper investigations into its safety, underlying molecular mechanisms, and treatment effects are necessary.

## Abbreviations

PDT: photodynamic therapy
SD-OCT: spectrum domain optical coherence tomography
FFA: fundus fluorescein angiography
MRI: magnetic resonance imaging
